# Exosomes derived from Wharton's jelly of human umbilical cord mesenchymal stem cells reduce osteocyte apoptosis in glucocorticoid-induced osteonecrosis of the femoral head in rats via the miR-21-PTEN-AKT signalling pathway

**DOI:** 10.7150/ijbs.32262

**Published:** 2019-07-20

**Authors:** Ming-jie Kuang, Ying Huang, Xi-ge Zhao, Rui Zhang, Jian-xiong Ma, Da-chuan Wang, Xin-long Ma

**Affiliations:** 1 Department of Orthopedics, Tianjin Hospital, Tianjin 300211, People's Republic of China; 2 Department of Orthopedics, The Provincial Hospital Affiliated to Shandong University, Shandong 250014, China

**Keywords:** Wharton's jelly of human umbilical cord mesenchymal stem cells, exosomes, GIONFH, apoptosis, AKT signalling pathway

## Abstract

*Purpose*: Glucocorticoid-induced osteonecrosis of the femoral head (GIONFH) is a common disease after long-term or high-dose glucocorticoid use. The pathogenesis of GIONFH is still controversial, and abnormal bone metabolism caused by glucocorticoids may be one of the important factors. Exosomes, owing to their positive effect on bone repair, show promising therapeutic effects on bone-related diseases. In this study, we hypothesised that exosomes reduce osteocyte apoptosis in rat GIONFH via the miR-21-PTEN-AKT signalling pathway.

*Methods*: To evaluate the effects of exosomes in GIONFH, a dexamethasone-treated or exosome-treated *in vitro* cell model and a methylprednisolone-treated *in vivo* rat model were set up. *In vitro*, a CCK-8 assay and 5-ethynyl-2′-deoxyuridine staining were performed to evaluate the proliferation of osteocytes. Further, a terminal deoxynucleotidyl transferase dUTP nick end labelling (TUNEL) assay, annexin V-fluorescein isothiocyanate-propidium iodide staining, and western blotting were conducted to evaluate the apoptosis of osteocytes. *In vivo*, we used micro-computed tomography and histological and immunohistochemical analyses to assess the effects of exosomes. Moreover, the mechanism of exosome action on osteocyte apoptosis through the miR-21-PTEN-AKT pathway was investigated by high-throughput RNA sequencing, fluorescence in situ hybridisation, luciferase reporter assays, and western blotting.

*Results*: High-throughput RNA sequencing results showed that the AKT signalling pathway was up-regulated in the exosome group. Quantitative PCR and western blotting confirmed that the relative expression of genes in the AKT pathway was up-regulated. Western blotting revealed that AKT activated by exosomes inhibited osteocyte apoptosis. RNA fluorescence in situ hybridisation and luciferase reporter assays were performed to confirm the interaction between miR-21 and PTEN. According to the experiment *in vivo*, exosomes prevented GIONFH in a rat model as evidenced by micro-computed tomography scanning and histological and immunohistochemical analyses.

*Conclusions*: Exosomes are effective at inhibiting osteocyte apoptosis (in MLO-Y4 cells) and at preventing rat GIONFH. These beneficial effects are mediated by the miR-21-PTEN-AKT signalling pathway.

## Introduction

Osteonecrosis of the femoral head (ONFH) is a pathological state with multiple possible aetiologies and characterised by a bone metabolism disorder^1^. Glucocorticoid-induced osteonecrosis of the femoral head (GIONFH) usually occurs after long-term or high-dose glucocorticoid use^2^. The annual number of patients increases steadily, moreover, among patients younger than 40 years, steroid use is the most prominent potential causative factor^3^. In China, a multi-centre epidemiological study on 6395 cases of ONFH has shown that 24.1% of cases are steroid induced^4^. Nonetheless, the pathogenesis of GIONFH is still not clear. Mont et al. have reported that fat embolism, vascular thrombosis, osteocyte and osteoblast apoptosis, and oxidative stress play an important part in GIONFH^5^. Glucocorticoid-driven disruption of normal bone metabolism may be the leading cause of GIONFH^6^.

The use of exosomes for treating GIONFH holds a remarkable potential as discovered in recent years. Exosomes derived from mesenchymal stem cells (MSCs)^7^ represent some of the most promising exosomes owing to their positive effect on bone repair. Studies have revealed the potential therapeutic effects of exosomes, e.g. antioxidant, anti-inflammatory, and anti-osteoporotic^8^. Nonetheless, little is known about their therapeutic effects on GIONFH. Zhang et al. have reported that exosomes derived from human platelet-rich plasma prevent apoptosis induced by glucocorticoid-associated endoplasmic-reticulum stress in rat ONFH, and the AKT-BAD-BCL-2 signalling pathway may play an important role according to that study^9^. In addition, exosomes from human synovium-derived MSCs hold good promise in rats with GIONFH^10^. All these studies have shown the osteogenesis caused by exosomes.

Human Wharton's jelly of umbilical cord mesenchymal stem cells (hWJ-MSCs) has stem cell properties and is easy to extract from the umbilical cord; hWJ-MSCs have a remarkable potential in the field of tissue regeneration. Nevertheless, no studies have addressed the anti-apoptosis effect of exosomes derived from hWJ-MSCs for GIONFH. In this study, high-throughput sequencing of RNA (RNA-seq) was performed to detect the genes differentially expressed under the influence of exosomes derived from hWJ-MSCs. A GIONFH rat model was created to investigate the pathogenesis of GIONFH. In addition, the protective effect of the exosomes derived from hWJ-MSCs was investigated, which was found to be mainly mediated by exosomal miR-21, which inhibits PTEN in osteocytes.

## Materials and methods

### Cell culture and treatments

hWJ-MSCs were cultured as previously reported^8^, and these cells were identified by flow cytometry. Positive markers (CD13, CD73, CD90, and CD105) and negative markers (CD34 and CD45) were analysed for identification of the cells (**Supplementary Fig.1**). Murine osteocyte-like MLO-Y4 cells were kindly provided by Prof. Lynda Bonewald (University of Missouri-Kansas City, Kansas City, MO, USA). MLO-Y4 cells (grown in culture dishes coated with 0.15 mg/mL rat tail type I collagen) were cultured in α-MEM (Hyclone, UK) with 2.5% of foetal bovine serum, 2.5% of calf serum, 100 U/mL penicillin, and 100 U/mL streptomycin at 5% CO_2_ and 37 °C^11^.

To investigate the effects of exosomes, MLO-Y4 cells were treated with dexamethasone (Dex), exosomes, or both. The concentration of Dex was 10 μM for 4 days for the CCK-8 analysis, and 10 μM for 24 h for 5-ethynyl-2′-deoxyuridine (EdU) staining. Next, 10 μM Dex was applied for 21 days in an osteogenic differentiation assay. Besides, 100 μM Dex was used for 24 h in an apoptosis experiment including a terminal deoxynucleotidyl transferase dUTP nick end labelling (TUNEL) assay, western blotting, and flow cytometry. The concentration of exosomes was 50 μg/mL.

### Exosome isolation, purification, and identification

Firstly, exo-free foetal bovine serum was prepared as previously reported^12^,^13^. The culture supernatant from hWJ-MSCs or MLO-Y4 cells was collected after 48 h cultivation. Then, the supernatant was centrifuged at 4000 rpm for 15 min to remove the cells, followed by filtration through a 0.22 μm filter to remove cell debris. Exosomes in the medium were precipitated with the exoEasy Maxi Kit (Qiagen) according to the manufacturer's instructions. The isolated exosomes were stored at -80 °C for later use.

Transmission electron microscopy (TEM) micrographs (Hitachi HT7700 transmission electron microscope, Tokyo, Japan) were analysed to determine the diameter of the exosomes.

The size distribution of exosomes was calculated by the Nanosizer^TM^ technology (Malvern, UK). The exosomes were diluted at the ratio of 1:1000 with 1 mL of PBS. The control medium and filtered PBS served as controls.

In addition, western blotting was performed to examine specific exosome biomarkers CD9, CD63, CD81, and TSG101.

### Exosome labelling with PKH-26

Exosome labelling with PKH-26 (Sigma) was performed following the manufacturer's instructions. Briefly, 100 µL of isolated exosomes were labelled with 40 µL of PKH-26, and then 500 µL of dilution buffer was added. The mixture was incubated in the dark for 20 min at room temperature. Next, 500 µL of 10% BSA was added to stop the staining reaction. Ultracentrifugation was conducted at 100000 × *g* for 1 h at 4 ºC, and then the supernatant was aspirated, and the exosomes were resuspended in PBS.

### RNA-seq

The high-throughput sequencing service was provided by CloudSeq Biotech (Shanghai, China). Transcriptome high-throughput sequencing and subsequent bioinformatic analysis were performed by Cloud-Seq Biotech (Shanghai, China). Briefly, total RNA was subjected to removal of rRNAs using the Ribo-Zero rRNA Removal Kit (Illumina, USA) following the manufacturer's instructions. RNA libraries were constructed by means of rRNA-depleted RNA samples with the TruSeq Stranded Total RNA Library Prep Kit (Illumina, USA) according to the manufacturer's instructions. The libraries were analysed for quality and quantified on a BioAnalyzer 2100 system (Agilent Technologies, USA). Next, 10 pM libraries were denatured into single-stranded DNA molecules, captured on Illumina flow cells, amplified in situ as clusters, and finally sequenced for 150 cycles on an Illumina HiSeq Sequencer according to the manufacturer's instructions.

### RNA fluorescence in situ hybridisation (FISH)

RNA FISH was conducted as previously reported^14^. The probe 5′- TCAACATCAGTCTGATAAGCTA-3′ labelled with Cy3 was synthesised from the sequence of miR-21-5p. The in situ hybridisation was then conducted according to the instructions of the FISH Detection Kit (Qiagen). Images were acquired with a confocal fluorescence microscope.

### MicroRNA (miRNA) transfection

MiR-21 mimics, inhibitors, and negative control (NC) (Sangon Biotech, Shanghai, China) were transfected into cells with Lipofectamine 3000 (Invitrogen) according to manufacturer's guidelines^15^. The working concentrations of miRNA mimics and mimic-NC were 50 nM, and those of miRNA inhibitors and inhibitor-NC were 100 nM. Next, 10 μM miR-21 Agomir or miR-21 Antagomir (Sangon Biotech, Shanghai, China) were injected intramuscularly once a week into GIONFH rats.

### A cell viability assay

We performed a Cell Counting Kit-8 assay (CCK-8) to estimate the cell proliferation rate. A total of 5000 MLO-Y4 cells per well were seeded in 96-well plates. We used different interventions including a control group, Dex group, Dex+exosomes group, and exosome group to assess cell proliferation. Absorbance was measured on an enzyme-linked immunosorbent assay reader (SpectraMax Plus 384, Molecular Devices, Sunnyvale, CA, USA) at 450 nm.

### A cell proliferation assay

EdU was employed to confirm the proliferation rate of the MLO-Y4 cells, and the results were evaluated by means of EdU-488 cells with the Flow Proliferation Cytometry Kit (Ribobio, Guangzhou, China) following the manufacturer's instructions. Briefly, the cells were seeded in a glass bottom plate at a density of 5 × 10^4^ cells/plate and cultured with the different interventions for 24 h. Then, the EdU working solution, mixed with 150 μL of a complete culture medium and 0.15 μL of EdU, was added into each well and incubated at 37°C for 3 h. After that, the cultured cells were harvested using trypsin-EDTA, washed three times with PBS, and fixed in 4% paraformaldehyde for 10 min. The fixed cells were neutralised with 2 mg/mL glycine and washed in PBS. After that, 0.4% Triton X-100 was used to permeabilise the cell membrane for 10 min, and the cells were washed three times with PBS. The labelled cells were resuspended in the Apollo staining solution, incubated for 10 min, and washed three times with PBS. The prepared cells were analysed on a Guava easy Cyte6HT-2L flow cytometer.

### A cell apoptosis assay

The Annexin V-FITC apoptosis detection kit (Miltenyi Biotec GmbH, Germany) was employed to estimate the effect of exosomes on MLO-Y4 cell apoptosis according to the manufacturer's instructions. Briefly, the cells were cultured in a 6-well plate. A total of 10^5^ cells were harvested, washed with PBS, and then resuspended in 100 μL of Annexin V binding buffer. Next, 10 μL of Annexin V-FITC and the cells were mixed well and incubated for 15 min in the dark at room temperature. Subsequently, the cells were washed by adding 1 mL of 1× binding buffer. Five microliters of the propidium iodide (PI) solution was immediately added prior to analysis by flow cytometry.

### A TUNEL assay

The TUNEL Kit (Roche, Switzerland) was used for detection and quantification of apoptosis at the cell level based on labelling of DNA strand breaks. Briefly, MLO-Y4 cells were seeded in a glass bottom plate at density 5 × 10^4^ cells and cultured during different interventions for 24 h. Next, 4% paraformaldehyde in PBS was applied to fix the cells (20 min incubation), and the cells were washed three time with PBS. We incubated the cells in 0.1% Triton X-100 with 0.1% sodium citrate to permeabilise the cell membrane for 2 min on ice, and then washed the cells three times with PBS. We incubated, fixed, and permeabilised the cells for 1 h with the TUNEL reaction mixture in a humidified atmosphere in the dark. The samples were analysed under a confocal fluorescence microscope at an excitation wavelength in the range of 450-500 nm.

### A western blotting assay and antibodies

The protein samples were extracted from cells or bone tissue by essentially the same procedures as described previously^9^. Briefly, cell or bone tissue lysates were diluted at a ratio of 1:5 with 5× loading buffer and heated at 95°C for 5 min. Next, 20 μL of the protein mixture was loaded onto the gel for electrophoresis. The proteins were separated by electrophoresis at 120 V for 1 h, and then blotted onto a polyvinylidene difluoride (PVDF) membrane (Merck-Millipore) for 60 min at 200 mA. After blocking for 3 h with 5% non-fat dried milk in Tris-buffered saline containing 0.1% Tween 20 (TBST), the PVDF membranes were incubated with primary antibodies at 4°C overnight. Horseradish peroxidase-labelled secondary antibodies were incubated with the membrane at 37°C for 1 h after the membrane was washed three times with TBST. Exposure machine Amersham Imager 600 was used to visualise the bands on the PVDF membrane.

The primary antibodies were anti-CD9 (Abcam, cat. # ab92726), anti-CD63 (Abcam, ab59479), anti-Total-AKT (Abcam, ab8805), anti-PTEN (Abcam, ab32199), anti-Runx2 (Abcam, ab76959), and anti-TSG101 (Abcam, 125011); anti-GAPDH (Proteintech, 10494-1-AP) and anti-CD81 (Proteintech, 14387-1-AP); anti-caspase-3 (Cell Signaling Technology, 9662), anti-Bcl-2 (Cell Signaling Technology, 3498), anti-Bax (Cell Signaling Technology, 14796), anti-phospho- (p-)AKT(Thr308) (Cell Signaling Technology, 13038), anti-p-AKT(Ser473) (Cell Signaling Technology, 4060), and an anti-p-Bad antibody (Cell Signaling Technology, 5284).

### Real-time reverse-transcriptase polymerase chain reaction (RT-PCR)

RT-PCR was performed as previously reported^14^. The TRIzol Reagent (Invitrogen) was used to extract total RNA. The PrimeScript RT reagent kit (TaKaRa, Japan) was employed for reverse transcription. RT-PCR analysis was performed with the SYBR Premix Ex Taq II kit (TaKaRa) and detection on a Roche LightCycler 480 sequence detection system. *GAPDH* served as a loading control for quantitation of mRNA.

### The animal model

All experimental and animal care procedures were approved by the Animal Research Ethics Committee of Tianjin Hospital and performed in accordance with the guidelines of the National Institutes of Health Guidelines for the Care and Use of Laboratory Animals. Eighteen 8-week-old female Sprague-Dawley rats weighing 300 ± 20 g were used. The rats were randomly and equally subdivided into five groups: (1) a control group (rats treated with PBS); (2) methylprednisolone (MPS) group; (3) MPS+exosomes group; (4) MPS+exosomes+miR-21 Antagomir group; and (5) the MPS+miR-21 Agomir group. To induce GIONFH in rats, pulsed high dose 100 mg/(kg•d) MPS was intramuscularly injected within the first week, and sustained low-dose 21 mg/(kg•d) MPS was administered after the next 4 weeks. In groups (3) and (4), 100 μg/d exosomes were injected intramuscularly. Five weeks later, the rats were euthanised, and the femoral heads were examined by micro-computed tomography (micro-CT) and histomorphological analysis.

### Micro-CT analysis

The femoral heads were scanned with an Inveon micro PET/CT manufactured by Siemens (Berlin, Germany). Briefly, we performed micro-CT to scan the femur from the femoral head to the femoral condyle. Then, three-dimensional structures were reconstructed on the Inveon analysis workstation. The cancellous bone region of the femoral head was chosen as a region of interest for quantitative analysis. The bone volume/total volume ratio (BV/TV), trabecular thickness (Tb.Ts), trabecular number (Tb.N), and trabecular separation (Tb.Sp) were calculated on the Inveon analysis workstation.

### Haematoxylin and eosin (HE) and immunohistochemical (IHC) staining

The collected femoral heads were fixed with paraformaldehyde for a week. The samples were embedded in paraffin and cut into 5 µm sections, deparaffinised in xylene, rehydrated in a graded series of ethanol solutions, and rinsed in distilled water. HE staining was performed for histological observation.

The osteogenesis of bone tissue around the femoral heads was assessed by IHC staining. The anti-Runx2 antibody was applied for IHC staining to evaluate the osteogenesis effect.

### Statistical analysis

All these experiments were repeated three times. The data are shown as means ± standard deviation (SD). Means of multiple groups were compared by one-way analysis of variance (with Fisher's least significant difference [LSD] test). Statistical analysis was conducted in SPSS 20.0 software (IBM Corp., Armonk, NY, USA). Data with P values <0.05 were considered statistically significant.

## Results

### Characterisation of hWJ-MSC-derived exosomes (hWJ-MSC-Exos)

**Figure 1:**
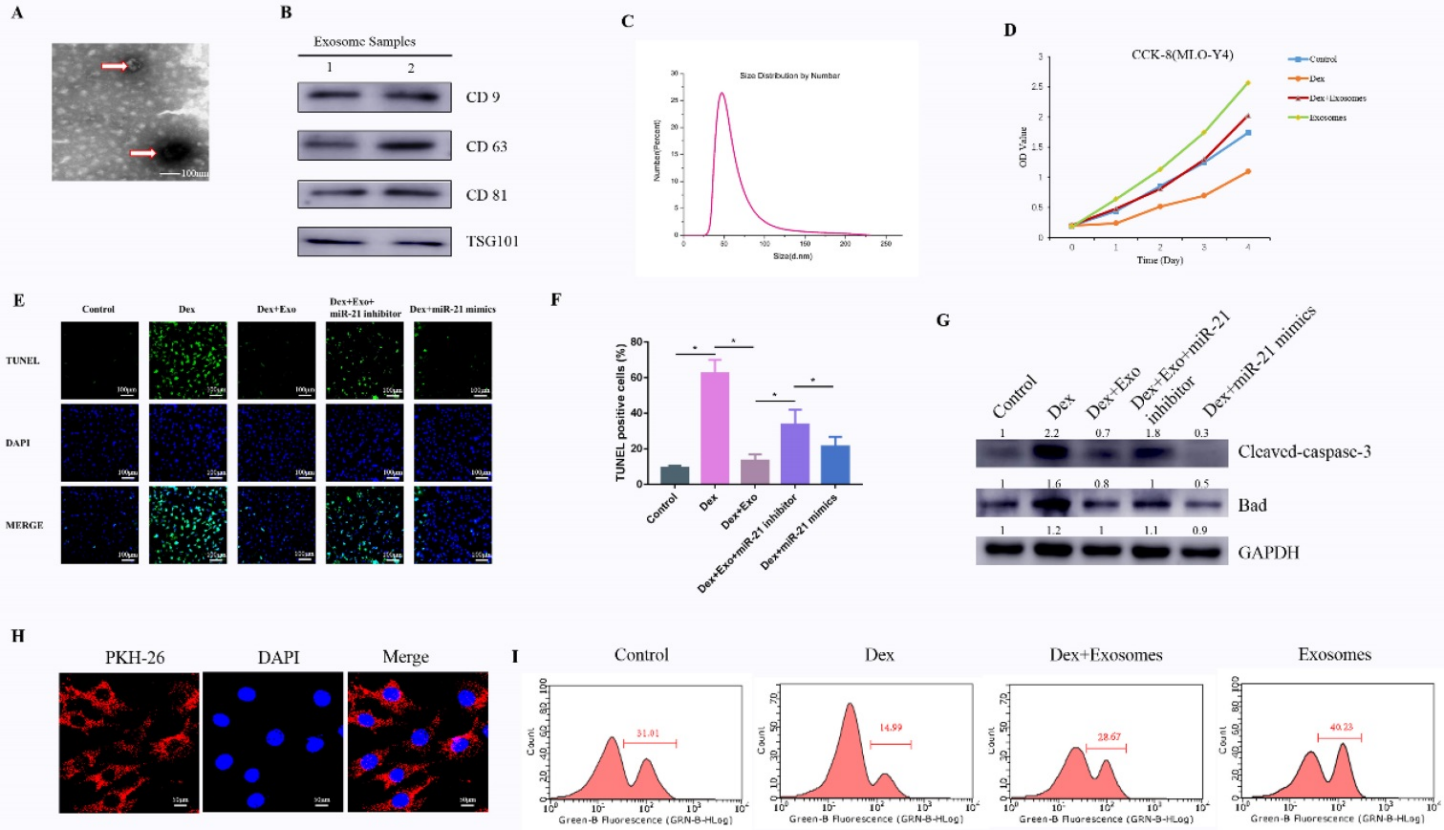
** (A)** Morphology of hWJ-MSC-Exos identified by TEM. **(B)** Western blotting analysis of the surface biomarkers CD9, CD63, CD81, and TSG101. **(C)** Particle size distribution of hWJ-MSC-Exos as measured by DLS. **(D)** The cell proliferation ability of MLO-Y4 cells treated with Dex and with or without hWJ-MSC-Exos. **(E)** The TUNEL assay was performed to evaluate the apoptosis of MLO-Y4 cells. **(F)** The percentage of TUNEL-positive cells was calculated in ImageJ. **(G)** Western blotting was performed to detect the expression of apoptosis-related proteins including caspase 3, cleaved caspase 3, BCL-2, and BAX. **(H)** hWJ-MSC-Exos were labelled with PKH-26, and endocytosis of hWJ-MSC-Exos was observed under a confocal microscope. **(I)** The cell proliferation ability of MLO-Y4 cells was evaluated by the EdU assay.

To comprehensively characterise the purified nanoparticles derived from hWJ-MSCs, TEM, dynamic light scattering (DLS) analysis, and western blotting were conducted. TEM showed that hWJ-MSC-Exos had a round shape with lipid membrane structure and a size ranging from 30 to 100 nm (**Fig. 1A**). Western blotting revealed that hWJ-MSC-Exos were positive for the characteristic exosomal surface markers including CD9, CD63, CD81, and TSG101 (**Fig. 1B**). The DLS measurements revealed that the approximate size of these particles varied between 30 and 100 nm (**Fig. 1C**). In addition, the exosomes were labelled by PKH26, and hWJ-MSC-Exos endocytosis was detected in MLO-Y4 cells (**Fig. 1H**).

### Effects of hWJ-MSC-Exos on the proliferation of cells treated with a GC *in vitro*

To investigate the effects of exosomes on cell proliferation at high concentrations of a GC, MLO-Y4 cells were cultured in a conditioned medium supplemented with Dex (10 μM) with or without exosomes for CCK-8 and EdU assays. The results of the CCK-8 assay revealed that Dex treatment reduced the proliferative capability of cells, whereas exosomes increased the proliferation rate of MLO-Y4 cells. Moreover, the Dex-induced down-regulation of proliferation was attenuated by the exosomes (**Fig. 1D and Supplementary Fig.3**). To confirm the ability of exosomes to promote proliferation, the EdU assay was performed (**Fig. 1I**). The result was the same as in the CCK-8 assay.

### Effects of hWJ-MSC-Exos on cell apoptosis induced by GCs *in vitro*

Cell apoptosis testing was carried out by the TUNEL assay and western blotting of apoptosis-related proteins. Firstly, the anti-apoptosis effects of exosomes in the early stage were evaluated by the TUNEL assay. The results meant that the number of apoptotic cells in the Dex group was notably higher compared to the control group, whereas the Dex-induced apoptotic effect was reversed as a result of treatment of MLO-Y4 cells with exosomes. When transfected with miR-21 inhibitor in Dex+exosomes group, apoptotic cells were higher compared to the Dex+exosomes group. When transfected with miR-21 mimics in Dex group, apoptotic cells decreased compared to the Dex group (**Fig. 1E and 1F**).

Next, western blotting of MLO-Y4 cell lysates indicated that cleaved caspase 3 and BAX were activated after 24 h of exposure to Dex, and these changes were attenuated by co-treatment with exosomes when compared to the control group. In addition, when transfected with miR-21 inhibitor in Dex+exosomes group, the expression of cleaved caspase 3 and BAX were higher compared to the Dex+exosomes group. When transfected with miR-21 mimics in Dex group, the expression of cleaved caspase 3 and BAX decreased compared to the Dex group (**Fig. 1G**).

### Analysis of functional attributes related to changes in hWJ-MSC-Exos: alterations of the expression of gene sets and signalling pathways

**Figure 2:**
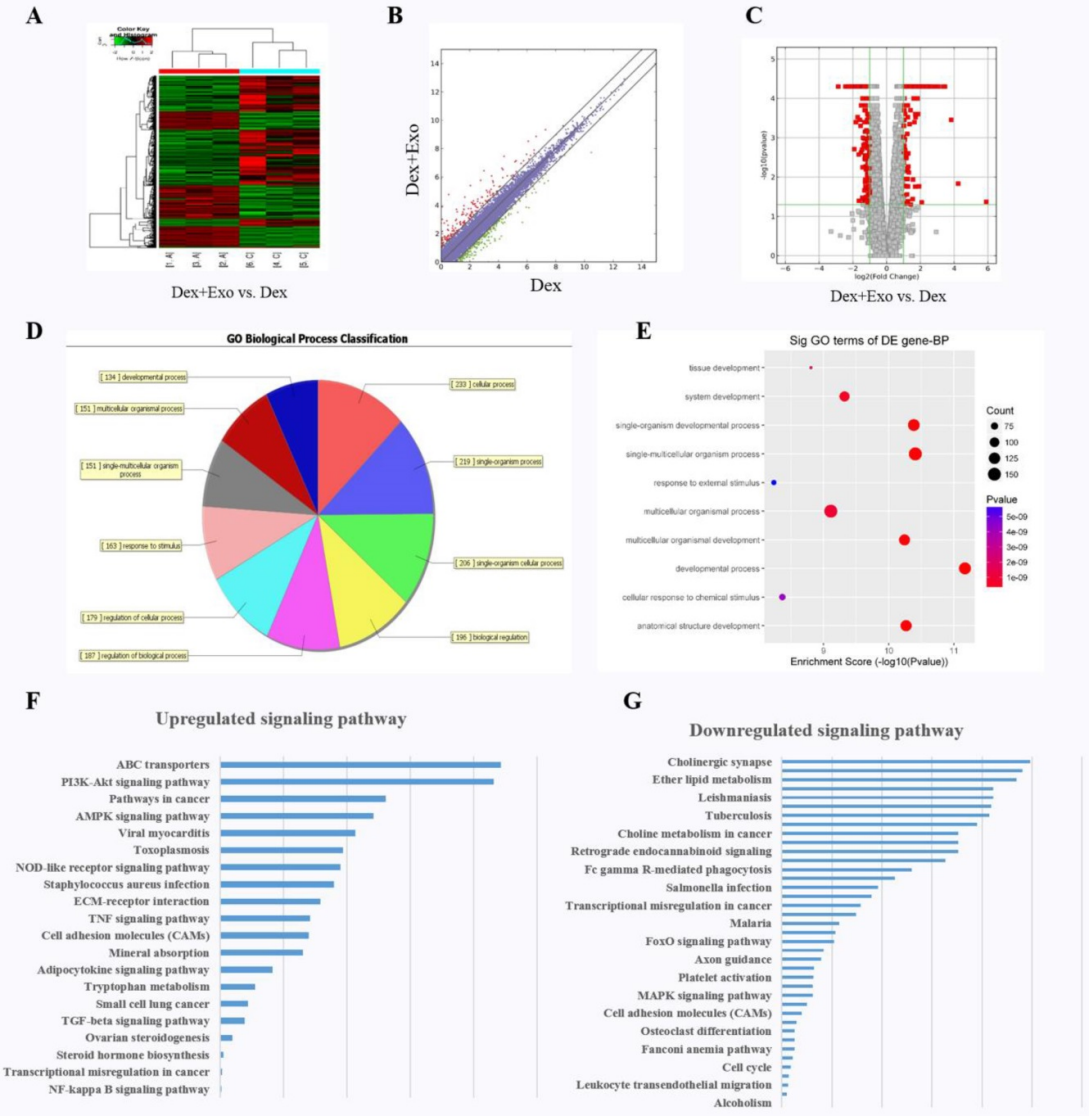
The heat map **(A)**, scatter plot **(B)**, and volcano plot **(C)** of RNA-seq between groups 'Dex' and 'Dex with hWJ-MSC-Exos'. **(D)** Gene Ontology biological process classification of the gene sets from RNA-seq. **(E)** Significant Gene Ontology terms enriched in the plot figure. **(F)** Up-regulated signalling pathways and **(G)** down-regulated signalling pathways enriched in the set of differentially expressed genes.

We explored the molecular biological mechanism of hWJ-MSC-Exos' positive action on cell proliferation and the inhibition of apoptosis. RNA-seq was performed to characterise the gene expression in MLO-Y4 cells. Finally, a total of 306 targets were found to be up-regulated in the Dex+Exo group, whereas 414 genes were down-regulated; the results are illustrated via a heat map (**Fig. 2A)**, scatter plot (**Fig. 2B)**, and volcano plot (**Fig. 2C**). The biological process categories, cellular component categories, and molecular function categories revealed that the exosomes play a key role in metabolic processes (**Fig. 2D and 2E**). To evaluate the functional features of exosome-affected gene expression, we performed KEGG pathway enrichment analysis, and the AKT signalling pathway was found to be up-regulated when MLO-Y4 cells were cultured with exosomes (**Fig. 2F and 2G**).

### hWJ-MSC-Exos rescued osteocytes from GC-induced apoptosis through the AKT pathway

**Figure 3:**
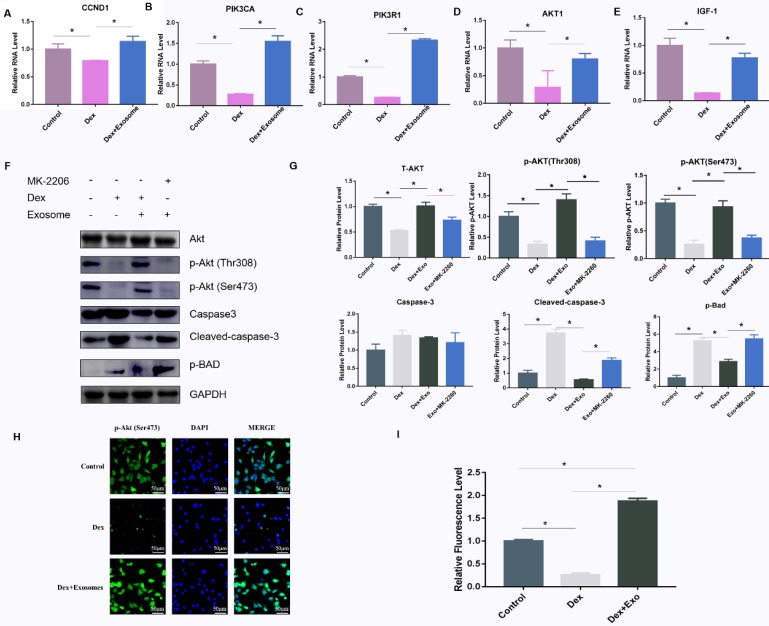
** (A-E)** The expression of genes including *CCND1*, *PIK3CA*, *PIK3R1*, *AKT1*, and *IGF-1* among the AKT signalling pathway gene sets was verified by RT-PCR. **(F)** Western blotting was performed to evaluate the protein amounts of AKT, p-AKT, caspase 3, cleaved caspase 3, and p-BAD. **(G)** The quantitative analysis of western blotting data in ImageJ. **(H)** Immunofluorescence staining was then performed to examine the amount of p-AKT in MLO-Y4 cells treated with Dex or exosomes. **(I)** Fluorescence intensity was quantified by means of ImageJ.

To verify the mechanisms of action of exosomes on GC-induced osteocyte apoptosis, real-time PCR was carried out to investigate the relevant genes including *CCDN1*, *PIK3R1*, *PIK3CA*, *AKT1*, and *IGF1* in the PI3K-AKT signalling pathway. The results showed that *AKT1*, *PIK3CA*, *PIK3R1*, *IGF1*, and *CCND1* were up-regulated via different patterns in Dex-treated cells **(Fig. 3A-E)**. In addition, western blotting was performed to further verify the protein expression in the PI3K-AKT signalling pathway, and the AKT inhibitor MK-2206 was used to investigate the expression of related genes. The results implied that high-dose GC activated BAD expression by inhibiting the phosphorylation of two key sites on AKT (Thr308 and Ser473), and then caspase 3 was cleaved, leading to apoptosis. When exosomes were added to the MLO-Y4 culture medium together with Dex, the AKT phosphorylation and p-BAD protein level decreased along with the cleaved-caspase-3 level. When the AKT inhibitor MK-2206 was added to the MLO-Y4 culture medium together with exosomes, the amount of p-AKT decreased, and p-BAD and cleaved caspase 3 were activated, leading to apoptosis **(Fig. 3F and 3G)**. In addition, the immunofluorescence assay was performed to confirm the positive effect on the activation of AKT expression** (Fig. 3H and 3I)**.

### Exosomal miR-21 regulates apoptosis through the PTEN-AKT signal pathway

**Figure 4:**
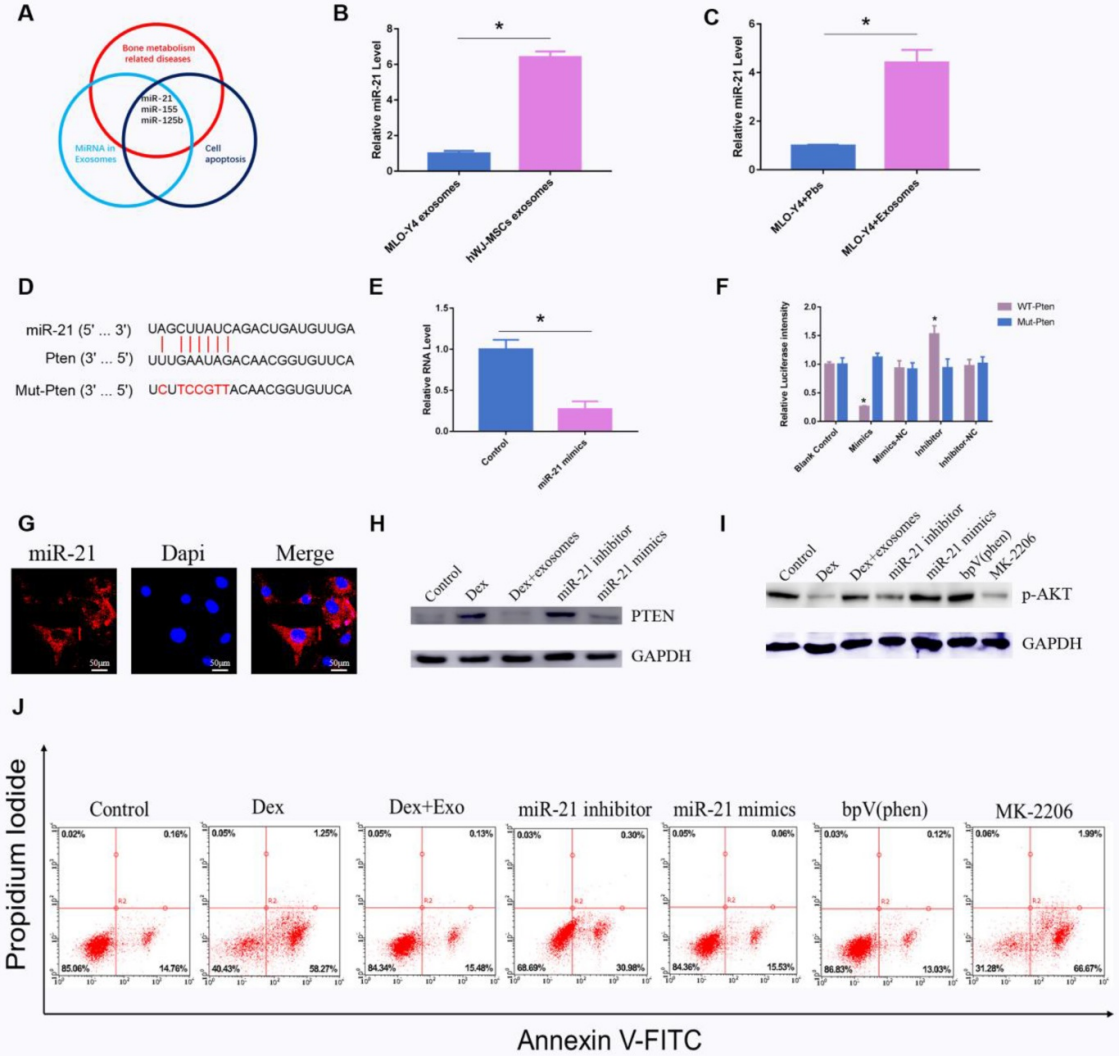
** (A)** Main reported miRNAs participating in apoptosis, bone-related diseases, and miRNAs present in exosomes; some miRNAs are multifunctional and are members of several groups, e.g. miR-21, miR-155, and miR-125b. **(B)** RT-PCR was performed to compare the level of miR-21 between hWJ-MSC-Exos and osteocyte exosomes. **(C)** RT-PCR was carried out to measure the level of miR-21 in osteocytes co-cultured with or without exosomes. **(D)** The binding site for miR-21 in *PTEN* mRNA. **(E)** The PTEN level when osteocytes were transfected with miR-21 mimics. **(F)** A luciferase reporter assay was performed to verify the interaction between miR-21 and PTEN. **(G)** FISH was conducted to identify the cell location of miR-21. **(H)** Western blotting was used to detect the interaction between miR-21 and PTEN. **(I)** Western blotting was conducted to evaluate the miR-21-PTEN-p-AKT axis. **(J)** The apoptosis of MLO-Y4 cells was assessed through annexin V-FITC/PI double staining with flow-cytometric analysis of the miR-21-PTEN-AKT signalling pathway.

As previously reported, exosomal miRNAs perform an important function in the regulation of apoptosis^16^. We screened all the articles that had been published in an electronic database with the search string 'miRNA AND exosomes AND apoptosis'. A total of 166 citations were found in the PubMed database. Among them, 44 review articles were excluded. Among the remaining 122 studies, miRNAs related to bone diseases were screened. We found that miR-21 is a potential regulator of apoptosis (**Fig. 4A**). Quantitative PCR revealed that miR-21 in hWJ-MSC-Exos was 6.11-fold up-regulated relative to the exosomes derived from MLO-Y4 cells (**Fig. 4B**). Next, we investigated the miR-21 level in MLO-Y4 cells when treated with hWJ-MSC-Exos or PBS, and the results indicated that the level of miR-21 was higher in the hWJ-MSC-Exo-treated group (**Fig. 4C**).

Firstly, we identified the cell location of miR-21, and FISH assays showed that miR-21 is mainly located in the cytoplasm (**Fig. 4G**). The target of miR-21 was also investigated by searching databases. TargetScan and mirDIP were employed to predict the target genes. The results showed that miR-21 interacts with *PTEN* mRNA (**Fig. 4D**), and the luciferase assay revealed too that miR-21 interacts with *PTEN* mRNA (**Fig. 4F**). Quantitative PCR indicated that miR-21 mimics inhibited the expression of PTEN (**Fig. 4E**). Western blotting confirmed that miR-21 mimics inhibited the expression of PTEN, whereas the miR-21 inhibitor promoted the expression of PTEN (**Fig. 4H**). It is known that PTEN is an inhibitor of the AKT signalling pathway. Our results showed that miR-21 mimics up-regulated p-AKT. BpV(phen), a PTEN inhibitor, increased the amount of p-AKT. MK-2206, an AKT inhibitor, down-regulated p-AKT (**Fig. 4I**). Apoptosis was also investigated, and the results were consistent with the above-mentioned data (**Fig. 4J**).

### hWJ-MSC-Exos affect GIONFH in the rat model

**Figure 5:**
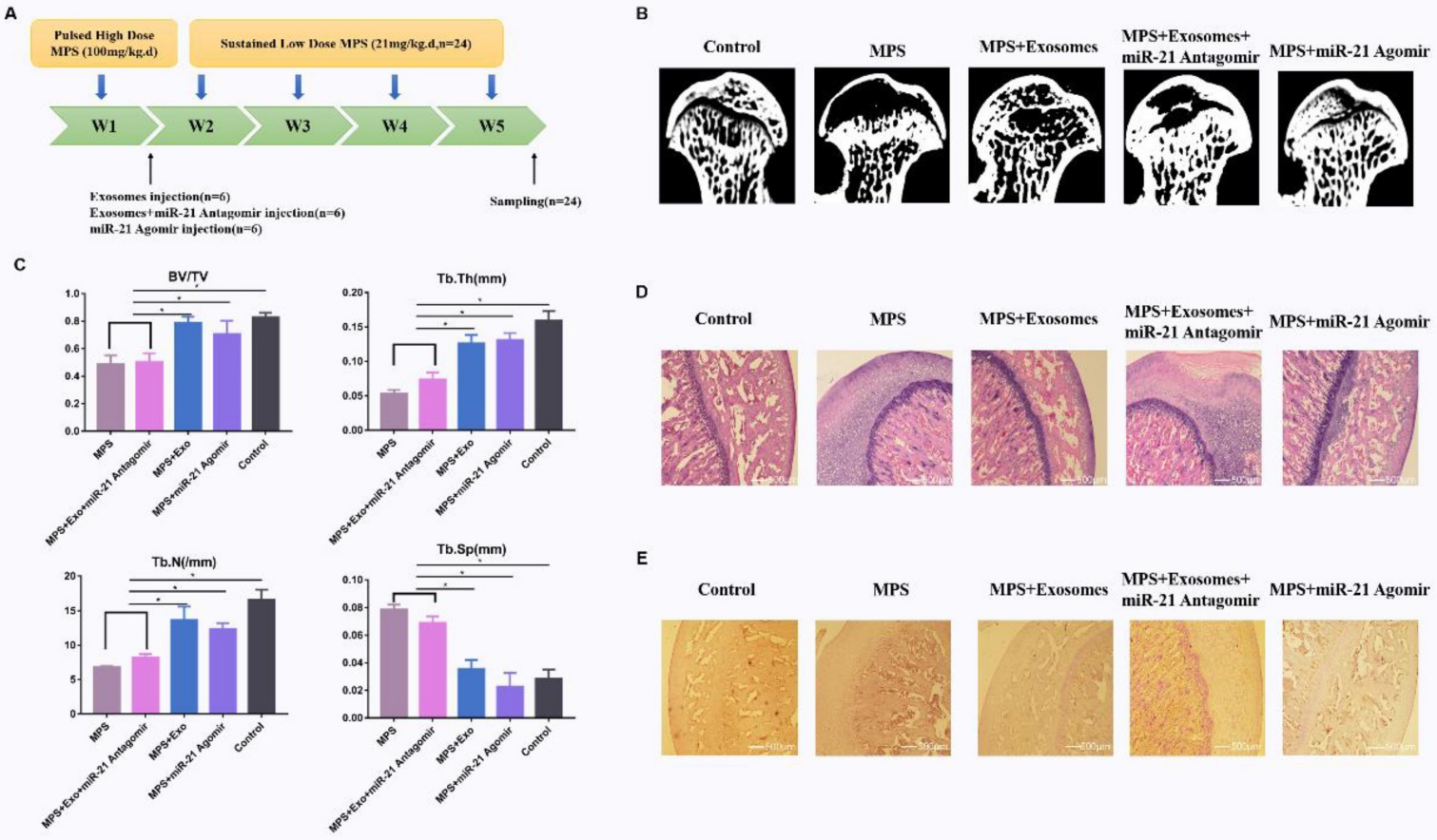
** (A)** The GIONFH rat model was created by means of MPS, and exosomes were tested for the treatment of GIONFH. **(B)** Micro-CT scanning was performed to assess the bone volume of the femoral head in the different rat groups. **(C)** HE staining of the femoral heads in rats receiving different treatments. **(D)** Quantitative analyses of trabecular thickness (Tb.Th), trabecular separation (Tb.Sp), bone volume per tissue volume (BV/TV), and trabecular number (Tb.N) in the different rat groups. **(E)** Immunohistochemical staining of p-AKT in samples from the different rat groups.

To investigate the effects of exosomes on MPS-induced ONFH, the rat model of ONFH was created by intramuscular injection of MPS with exosomes or an equal volume of saline **(Fig. 5A)**. Five weeks after the treatment, micro-CT was performed to qualitatively evaluate the bone tissues within the femoral head **(Fig. 5B)**. Microstructural parameters BV/TV, Tb.Th, and Tb.N in the MPS group were significantly worse when compared with the control and MPS+exosomes group, i.e. exosomes remarkably reversed the MPS-induced bone loss. Furthermore, Tb.Sp was significantly higher in the MPS group compared to the normal group, and this increase was profoundly attenuated by exosomes **(Fig. 5D)**.

The HE staining results meant that osteonecrosis was well detectable in the MPS group; moreover, the trabecular bone of the femoral head became sparser and was replaced by necrotic tissues. In contrast, the trabecular bone in the rats additionally treated with naringin was well arranged, and little trabecular bone was replaced by necrotic tissues. In addition, no osteonecrosis was observed in the normal group **(Fig. 5C)**. IHC staining for p-AKT revealed that AKT phosphorylation in femoral heads decreased in the MPS group, but this effect was reversed by exosomes **(Fig. 5E)**.

## Discussion

Osteocytes are the most abundant cells in the bone tissue and are important for the maintenance of bone strength and bone metabolism^17^. The apoptosis of osteocytes caused by GCs is one of the important mechanisms of GIONFH^18^. In our study, the inhibitory effect of hWJ-MSC-Exos on osteocyte apoptosis was found to be mediated by the miR-21-PTEN-AKT axis, whereas apoptotic proteins such as BAD and caspase 3 were inhibited. Therefore, hWJ-MSC-Exos exerted a strong anti-apoptotic effect in GIONFH.

Several studies have characterised the effects of GCs in GIONFH: they range between osteogenesis and osteoclastogenesis^19^-^21^. Although osteocytes are the most abundant cells in bone tissue, rare studies reported the mechanism of GC action on osteocytes. Weinstein et al.^22^ have reported that GCs decrease the production of osteoblasts, whereas accumulation of apoptotic osteocytes contributes to ONFH. In our study, GCs inhibited the proliferation of osteocytes and contributed to the apoptosis of osteocytes. hWJ-MSC-Exos showed a remarkable inhibitory effect on osteocyte apoptosis caused by GCs. In addition, we investigated the mechanism of hWJ-MSC-Exos' inhibitory action on osteocyte apoptosis and found that the miR-21-PTEN-AKT axis may be an effective target for the treatment of GIONFH.

The anti-apoptotic effect of exosomes has also been demonstrated in another study^23^. Xiao et al. have reported that cardiac progenitor cell-derived exosomal miR-21 has an inhibitory effect on the apoptosis pathway via down-regulation of PDCD4,^15^ and restoration of the miR-21-PDCD4 pathway using cardiac progenitor cell-derived exosomes may protect myocardial cells from oxidative-stress-related apoptosis. Wu et al. have reported that hWJ-MSC-Exos have profound alleviating effects on dextran sulphate sodium-induced inflammatory bowel disease and may exert their action by changing the ubiquitin modification level^24^. Nevertheless, no researchers have reported whether hWJ-MSC-Exos are effective in treating GIONFH. In our study, we found that hWJ-MSC-Exos promoted osteocyte proliferation and reversed the apoptotic effect caused by GCs. In addition, we verified the anti-apoptotic effect of hWJ-MSC-Exos by TUNEL staining, the annexin V-FITC/PI assay, and western blotting. All these findings indicate that hWJ-MSC-Exos can maintain the osteocyte number in GIONFH.

We also explored the relevant target genes via bioinformatic analysis and RNA-seq. We found that the AKT signalling pathway may be a key player. Genes in the AKT signalling pathway gene set such as *Ccdn1*, *Pik3ca*, *Pik3r1*, *Igf1,* and *Akt1* were up-regulated by the exosome treatment. GCs induced osteocyte apoptosis due to inhibition of AKT phosphorylation, and thus, BAD was phosphorylated and induced osteocyte apoptosis resulting in GIONFH, in agreement with previous research^9^. There is no doubt that hWJ-MSC-Exos strongly promote osteocyte proliferation and inhibit osteocyte apoptosis through the AKT signalling pathway.

In addition, we investigated the mechanism upstream of AKT activation. Recent studies showed that exosomal miR-21 participates in apoptosis^15^,^25^. In our study, we found that miR-21 was more abundant in hWJ-MSCs when compared with MLO-Y4 exosomes; this finding indicates that miR-21 is present in hWJ-MSCs. Quantitative PCR confirmed this result. Moreover, we found that miR-21 interacts with *PTEN* mRNA in a luciferase reporter assay and after western blotting. Moreover, miR-21 mimics inhibited the expression of PTEN, whereas the miR-21 inhibitor up-regulated PTEN. PTEN is an inhibitor of the AKT signalling pathway and suppresses the expression of AKT^26^. We verified the relation between PTEN and AKT using a PTEN inhibitor and AKT inhibitor and found that repression of PTEN increased AKT activation. Therefore, we demonstrated that exosomal miR-21 alleviates GIONFH through the PTEN-AKT signal pathway. A rat model of GIONFH was constructed here to verify the benefits of hWJ-MSC-Exos. The results of micro-CT, HE staining, and IHC staining all mean that hWJ-MSC-Exos are effective against GIONFH.

In summary, we characterised the inhibitory action of hWJ-MSC-Exos on osteocyte apoptosis. Moreover, these results for the first time show that the miR-21-PTEN-AKT signalling pathway plays a crucial role in the control of osteocyte apoptosis in GIONFH. Findings from this study will help clinical researchers to test hWJ-MSC-Exos in the treatment of GIONFH.

## Supplementary Material

Supplementary figures.

## References

[1] Mont MA, Cherian JJ, Sierra RJ, Jones LC, Lieberman JR (2015). Nontraumatic Osteonecrosis of the Femoral Head: Where Do We Stand Today? A Ten-Year Update. The Journal of bone and joint surgery American volume.

[2] Zheng L-Z, Wang J-L, Kong L, Huang L, Tian L, Pang Q-Q (2018). Steroid-associated osteonecrosis animal model in rats. Journal of Orthopaedic Translation.

[3] Fukushima W, Fujioka M, Kubo T, Tamakoshi A, Nagai M, Hirota Y (2010). Nationwide epidemiologic survey of idiopathic osteonecrosis of the femoral head. Clin Orthop Relat Res.

[4] Cui L, Zhuang Q, Lin J, Jin J, Zhang K, Cao L (2016). Multicentric epidemiologic study on six thousand three hundred and ninety five cases of femoral head osteonecrosis in China. International orthopaedics.

[5] Mont MA, Jones LC, Hungerford DS (2006). Nontraumatic osteonecrosis of the femoral head: ten years later. The Journal of bone and joint surgery American volume.

[6] Weinstein RS (2012). Glucocorticoid-induced osteonecrosis. Endocrine.

[7] Tkach M, Thery C (2016). Communication by Extracellular Vesicles: Where We Are and Where We Need to Go. Cell.

[8] Fan YP, Hsia CC, Tseng KW, Liao CK, Fu TW, Ko TL (2016). The Therapeutic Potential of Human Umbilical Mesenchymal Stem Cells From Wharton's Jelly in the Treatment of Rat Peritoneal Dialysis-Induced Fibrosis. Stem cells translational medicine.

[9] Tao SC, Yuan T, Rui BY, Zhu ZZ, Guo SC, Zhang CQ (2017). Exosomes derived from human platelet-rich plasma prevent apoptosis induced by glucocorticoid-associated endoplasmic reticulum stress in rat osteonecrosis of the femoral head via the Akt/Bad/Bcl-2 signal pathway. Theranostics.

[10] Guo S, Tao S, Yin W, Qi X, Sheng J, Zhang C (2016). Exosomes from Human Synovial-Derived Mesenchymal Stem Cells Prevent Glucocorticoid-Induced Osteonecrosis of the Femoral Head in the Rat. Int J Biol Sci.

[11] Kato Y, Windle JJ, Koop BA, Mundy GR, Bonewald LF Establishment of an osteocyte-like cell line, MLO-Y4 Journal of bone and mineral research: the official journal of the American Society for Bone and Mineral Research. 1997; 12: 2014-23.

[12] Peinado H, Aleckovic M, Lavotshkin S, Matei I, Costa-Silva B, Moreno-Bueno G (2012). Melanoma exosomes educate bone marrow progenitor cells toward a pro-metastatic phenotype through MET. Nature medicine.

[13] Su T, Xiao Y, Xiao Y, Guo Q, Li C, Huang Y Bone Marrow Mesenchymal Stem Cells-Derived Exosomal MiR-29b-3p Regulates Aging-Associated Insulin Resistance. 2019; 13: 2450-62.

[14] Zheng Q, Bao C, Guo W, Li S, Chen J, Chen B (2016). Circular RNA profiling reveals an abundant circHIPK3 that regulates cell growth by sponging multiple miRNAs. Nature communications.

[15] Xiao J, Pan Y, Li XH, Yang XY, Feng YL, Tan HH (2016). Cardiac progenitor cell-derived exosomes prevent cardiomyocytes apoptosis through exosomal miR-21 by targeting PDCD4. Cell death & disease.

[16] Zhang J, Li S, Li L, Li M, Guo C, Yao J (2015). Exosome and Exosomal MicroRNA: Trafficking, Sorting, and Function. Genomics, Proteomics & Bioinformatics.

[17] Ferron M, Wei J, Yoshizawa T, Del Fattore A, DePinho RA, Teti A (2010). Insulin signaling in osteoblasts integrates bone remodeling and energy metabolism. Cell.

[18] Lee NK, Sowa H, Hinoi E, Ferron M, Ahn JD, Confavreux C (2007). Endocrine regulation of energy metabolism by the skeleton. Cell.

[19] Shi C, Qi J, Huang P, Jiang M, Zhou Q, Zhou H (2014). MicroRNA-17/20a inhibits glucocorticoid-induced osteoclast differentiation and function through targeting RANKL expression in osteoblast cells. Bone.

[20] Tokuyama N, Hirose J, Omata Y, Yasui T, Izawa N, Matsumoto T (2015). Individual and combining effects of anti-RANKL monoclonal antibody and teriparatide in ovariectomized mice. Bone Reports.

[21] Conradie MM, Cato AC, Ferris WF, de Wet H, Horsch K, Hough S (2011). MKP-1 knockout does not prevent glucocorticoid-induced bone disease in mice. Calcified tissue international.

[22] Weinstein RS, Jilka RL, Parfitt AM, Manolagas SC (1998). Inhibition of osteoblastogenesis and promotion of apoptosis of osteoblasts and osteocytes by glucocorticoids. Potential mechanisms of their deleterious effects on bone. The Journal of clinical investigation.

[23] Lotvall J, Hill AF, Hochberg F, Buzas EI, Di Vizio D, Gardiner C (2014). Minimal experimental requirements for definition of extracellular vesicles and their functions: a position statement from the International Society for Extracellular Vesicles. J Extracell Vesicles.

[24] Wu Y, Qiu W, Xu X, Kang J, Wang J, Wen Y (2018). Exosomes derived from human umbilical cord mesenchymal stem cells alleviate inflammatory bowel disease in mice through ubiquitination. Am J Transl Res.

[25] Zheng P, Chen L, Yuan X, Luo Q, Liu Y, Xie G (2017). Exosomal transfer of tumor-associated macrophage-derived miR-21 confers cisplatin resistance in gastric cancer cells. Journal of Experimental & Clinical Cancer Research: CR.

[26] Chalhoub N, Baker SJ (2009). PTEN and the PI3-kinase pathway in cancer. Annual review of pathology.

