# Supplementary material for: Exosomes derived from Wharton's jelly of human umbilical cord mesenchymal stem cells reduce osteocyte apoptosis in glucocorticoid-induced osteonecrosis of the femoral head in rats via the miR-21-PTEN-AKT signalling pathway
Source: Int J Biol Sci. 2019 Jul 20;15(9):1861–71. doi: 10.7150/ijbs.32262 (PMC6743291; doi:10.7150/ijbs.32262)
Supplement: Supplementary file 1 — Supplementary figures. [file ijbsv15p1861s1.pdf]

Supplemental Fig. 1: A: The cellular morphology of hWJ-MSCs. B: Alizarin red staining of hWJ-MSCs. C: Alcian blue staining of hWJ-MSCs. D: Oil red O staining of hWJ-MSCs. E: The positive markers, including CD13, CD73, CD90, and CD105, and negative markers CD34 and CD45 of hWJ-MSCs using flow cytometry.

Supplemental Fig. 2: The statistic results of CCK-8 analysis.

Supplemental Fig. 3: The statistic results of Edu staining.

Supplemental Fig. 4: The statistic results of annexin V-FITC/PI double staining with flow-cytometric analysis.

Sup Fig.1

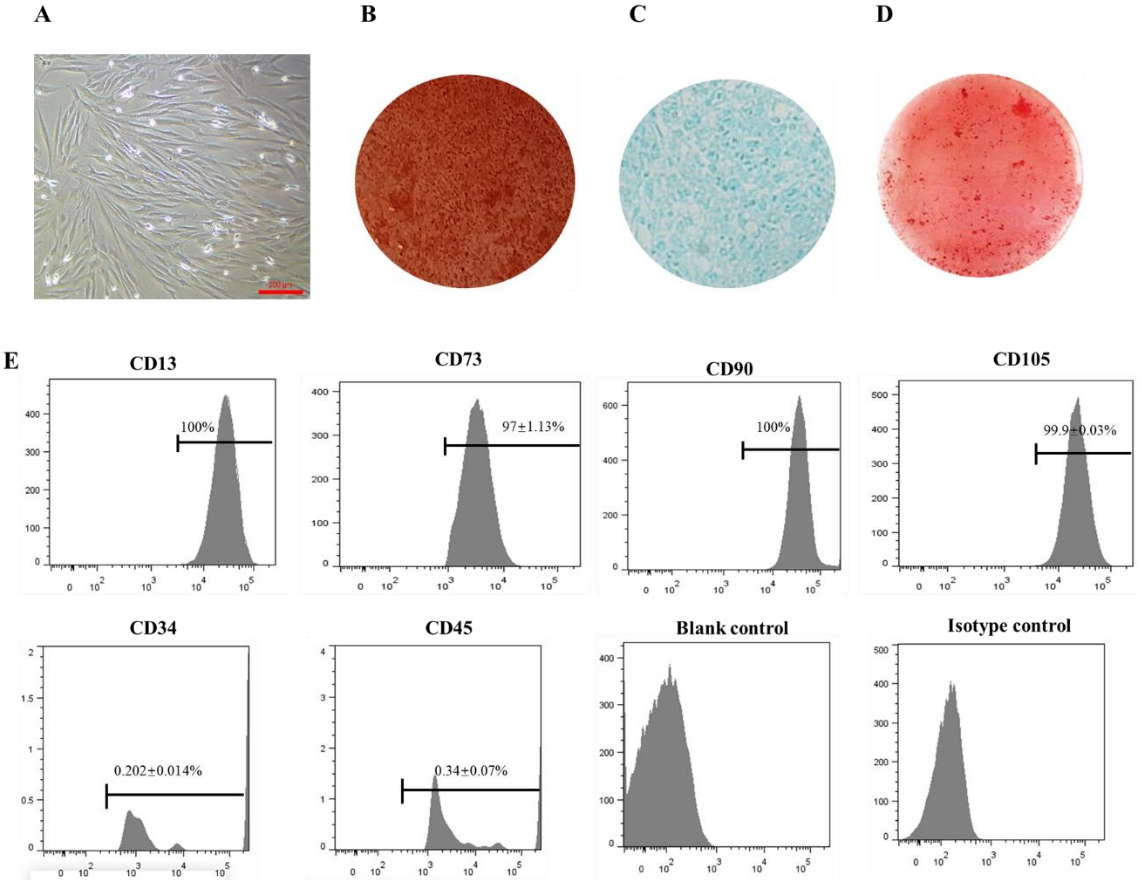
A
B
C
D
E
CD13
100%
CD73
97±1.13%
CD90
100%
CD105
99.9±0.03%
CD34
0.202±0.014%
CD45
0.34±0.07%
Blank control
Isotype control

Sup Fig.2

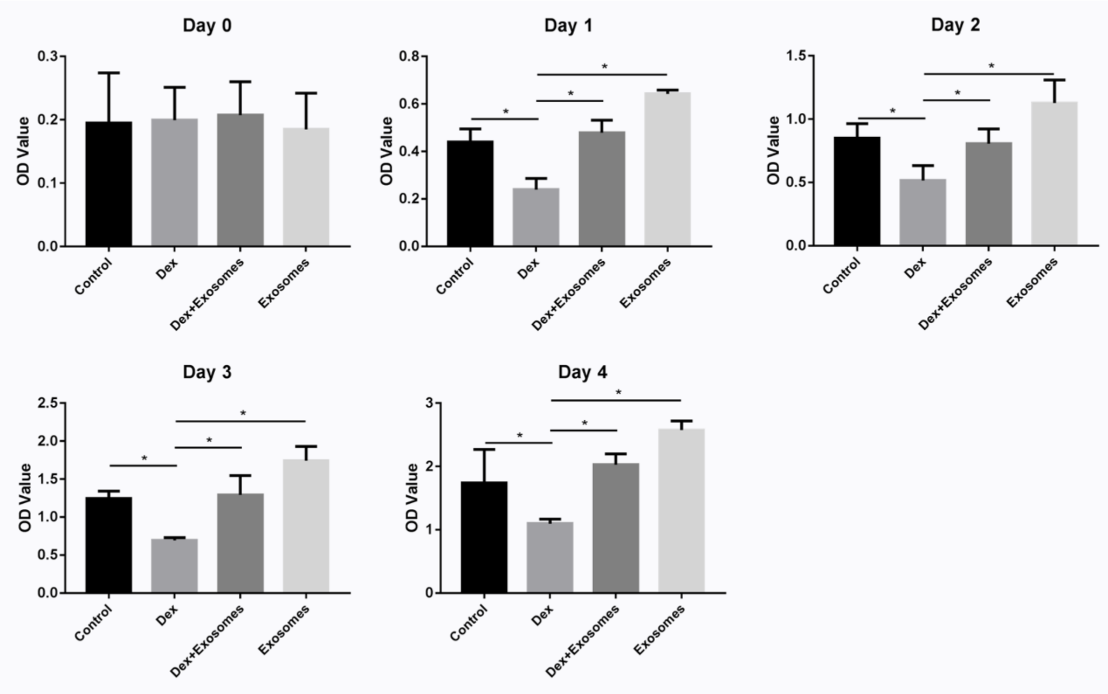

Day 0
Day 1
Day 2
Day 3
Day 4
OD Value
Control
Dex
Dex+Exosomes
Exosomes
*

Sup Fig.3

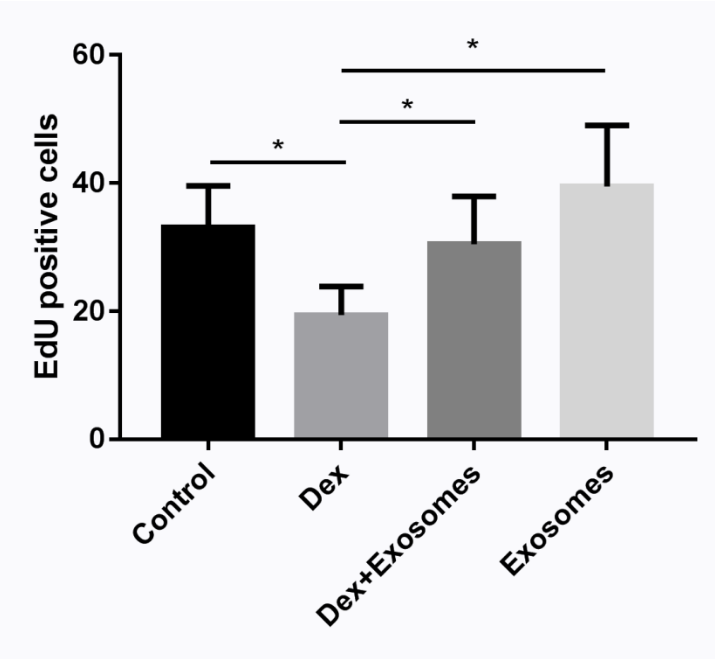

60
40
20
0
EdU positive cells
*
*
*
Control
Dex
Dex+Exosomes
Exosomes

Sup Fig.4

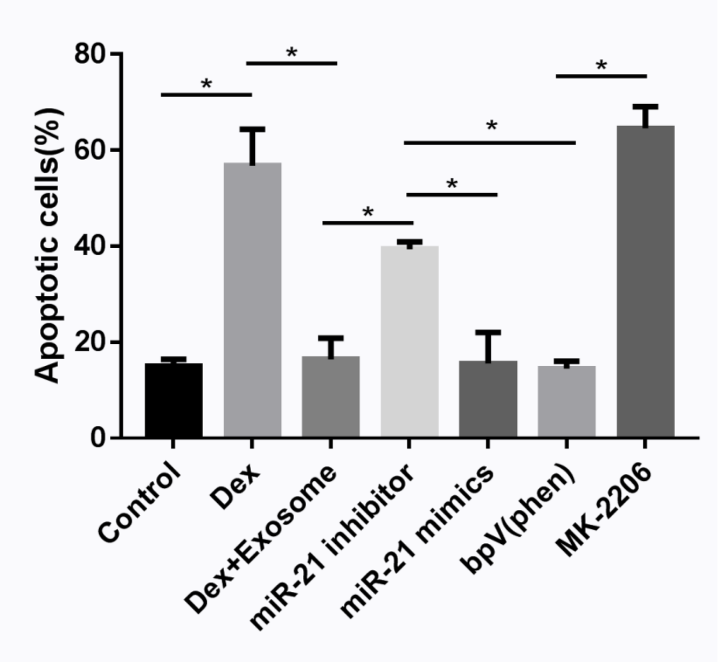

Apoptotic cells(%)
80
60
40
20
0
*
*
*
*
*
*
Control
Dex
Dex+Exosome
miR-21 inhibitor
miR-21 mimics
bpV(phen)
MK-2206
